# When survey science met web tracking: Presenting an error framework for metered data

**DOI:** 10.1111/rssa.12956

**Published:** 2022-11-06

**Authors:** Oriol J. Bosch, Melanie Revilla

**Affiliations:** ^1^ Department of Methodology The London School of Economics and Political Science London UK; ^2^ Research and Expertise Centre for Survey Methodology (RECSM) Universitat Pompeu Fabra Barcelona Spain

**Keywords:** digital trace data, error framework, metered data, passive data, total survey error, web‐tracking

## Abstract

Metered data, also called web‐tracking data, are generally collected from a sample of participants who willingly install or configure, onto their devices, technologies that track digital traces left when people go online (e.g., URLs visited). Since metered data allow for the observation of online behaviours unobtrusively, it has been proposed as a useful tool to understand what people do online and what impacts this might have on online and offline phenomena. It is crucial, nevertheless, to understand its limitations. Although some research have explored the potential errors of metered data, a systematic categorisation and conceptualisation of these errors are missing. Inspired by the Total Survey Error, we present a Total Error framework for digital traces collected with Meters (TEM). The TEM framework (1) describes the data generation and the analysis process for metered data and (2) documents the sources of bias and variance that may arise in each step of this process. Using a case study we also show how the TEM can be applied in real life to identify, quantify and reduce metered data errors. Results suggest that metered data might indeed be affected by the error sources identified in our framework and, to some extent, biased. This framework can help improve the quality of both stand‐alone metered data research projects, as well as foster the understanding of how and when survey and metered data can be combined.

## 1 INTRODUCTION

### 1.1 Definitions and main issues

Given the widespread adoption of the Internet, it is becoming vital to better understand what people do online and what impact this has on online and offline phenomena. This requires high‐quality data regarding people's online behaviours. Although surveys are one of the most used methods for collecting data in the social sciences (Sturgis & Luff, 2021), it can be complex for participants to accurately remember and report their behaviours through different devices and contexts. Besides, the type of data that is collectable with surveys, as well as its granularity, is inherently limited (e.g., we cannot ask thousands of questions on a single questionnaire, every day). Therefore, in recent years there has been an increase in the use of digital trace data to directly observe what people do online (Breuer et al., 2020).

A prominent strategy to collect traces about the web browsing and app behaviour of individuals has been to use digital tracking solutions (Christner et al., 2022). These solutions, called meters (Revilla et al., 2021), are a heterogeneous group of tracking technologies that can be installed, upon agreement, by participants on their browsing devices. Meters then allow for a variety of traces left by participants when interacting with their devices online to be tracked. Depending on the characteristics of the technology, different traces can be collected. For instance, the URLs or apps visited, the terms used in search engines or the content that participants have been exposed to (e.g., HTML information). A variety of terms have been used in the literature to refer to this resulting data, for example, ‘web‐tracking data’, ‘web log data’ and ‘digital trace data’ (e.g., Bach et al., 2019; Cardenal et al., 2019; Cid, 2018; Dvir‐Gvirsman et al., 2016). Although data coming from meters might fall under the umbrella of these broad terms, following Revilla et al. (2017), we use the term *metered data*, which describes the exact data collection procedure.

By directly capturing the digital traces created by participants when interacting with their devices online, data free of recall errors and memory limitations can be captured, with a granularity not achievable by surveys (Revilla, 2022). This data can be used to measure behavioural concepts of interest, potentially bypassing some of the challenges faced by self‐reports when measuring online behaviours to make inferences about theoretical concepts for finite populations. Non‐behavioural concepts like attitudes might also be measurable with metered data, although there may be fewer benefits.

Albeit limited attention has been paid to metered data errors when used to draw statistical inferences for finite populations, some research has warned about potential errors (Jürgens et al., 2019; Revilla et al., 2017). Indeed, a recent report from the Pew Research Center (2016) concluded that ‘there are still too many pitfalls to rely on [metered data] (…) [metered data] does not, at present, seem well suited for high‐level estimates of news consumptions’. However, a systematic categorisation and conceptualisation of these errors have yet to be developed. Thus, in this paper, we propose a Total Error framework for digital traces collected with Meters (TEM). Total Error is a paradigm used to refer to all the sources of bias and variance that may affect the accuracy and efficiency of data (Lavrakas, 2008). When operationalised as a framework, the Total Error conceptualises and categorises the different sources of error allowing for the understanding of the data collection and analysis process, as well as the identification and estimation of potential errors, their effects on estimates and how to minimise them.

### 1.2 Goals and contribution

The two main goals of the TEM framework are to (1) describe the data generation and analysis process of metered data and (2) document all error sources that can affect metered data when they are used to conduct inferential statistics (both univariate and multivariate). To this end, we adapt the Total Survey Error framework (TSE, Groves et al., 2009) for metered data, assuming that the error components presented in the TSE framework can also be found in metered data. Hence, instead of creating a completely new framework for metered data, we start from the TSE framework and modify it to the specific error generating processes and error causes of metered data. Consequently, the TEM can be used by researchers from different backgrounds. This framework provides a common understanding of how to choose the best design options for metered data projects and how to catalogue the potential errors affecting them. For projects integrating both metered and survey data collection (sometimes referred to as *Smart Surveys*; see Ricciato et al., 2020), the TEM can also help to make better‐informed decisions while planning when and how to supplement or replace survey data with metered data.

The TEM framework enriches the current landscape of Total Error frameworks, which have been developed for different types of digital trace data sources. Indeed, previous frameworks (see Section 2.2) have focused on secondary data, which are usually called ‘found’ or ‘organic’ digital trace data and which are not designed for research, like the data coming from online platforms (e.g., Sen et al., 2021). Instead, we propose a framework adapted to design‐based digital data, mapping in detail the specific error sources produced when tracking individuals' online behaviours using the heterogeneous group of technologies identified as meters. Furthermore, we illustrate how the TEM framework can help plan metered data collection while minimising errors, using a case study: the Triangle of Polarisation, Political Trust and Political Communication (TRI‐POL) project (https://www.upf.edu/web/tri‐pol). This project combined a cross‐national longitudinal survey and metered data collection. We show how the TEM was implemented in the design stage to document, quantify and minimise (when possible) potential error sources affecting the metered data. We also present empirical evidence about the prevalence of some of the error sources in the TRI‐POL data sets, and how these might affect the quality of metered data.

## 2 BACKGROUND

### 2.1 Distinctive aspects of metered data

As a design‐based digital data source, metered data differ from found data sources in two key design aspects: the *data collection* and *sampling* approaches.

Regarding found data, the nature and quality of the data are heavily limited by the original purpose of the traces (e.g., data from Twitter can only be obtained as Twitter intends and allows to), and the approaches available to download them (APIs, web scrapping, partnering with companies). Researchers have little control over this. Metered data, conversely, are produced by specific tracking solutions that capture the traces that participants generate when interacting with their devices and online services. Although the feasibility of collecting these traces is limited to some extent by the technological capabilities and the ‘friendliness’ of the different operating systems and online platforms towards tracking solutions (e.g., iOS terms and conditions do not allow tracking apps), meters can be considered as the main factor shaping what data can be collected, as well as their characteristics. Many different technological approaches have been used to collect metered data (see Christner et al., 2022  and Breuer et al., 2020 for in‐depth reviews), which can be broadly grouped into four categories:

1. Apps that passively and continuously track information from a device and its browser(s).
2. Plug‐ins that passively and continuously collect web browsing history and other device and browsing information.
3. Plug‐ins that collect the available web browsing history at a given point in time, but without continuously tracking the device/browser activity.
4. Manually configured proxies that send all internet connections made by a device through a network (e.g., WIFI at home) to a server set by the researcher. This information is automatically stored.

These different tracking solutions vary in many aspects, but two are particularly important from a data quality perspective. First, not all the different tracking technologies can collect the same type of information nor with the same frequency, granularity and precision. For instance, if data must be collected after participants install the technology, using the second category (a plug‐in that collects available browsing history) would not be ideal. Second, tracking solutions differ according to the devices (PC or mobile), Operating Systems (OSs; e.g., Android or iOS for mobile devices) and browsers (e.g., Chrome or Firefox) on which they can be installed. Hence, tracking solutions impact both who is tracked and how well they are tracked. The Supplementary Online Material (SOM) 1 of Data S1 summarises the capabilities and limitations of the tracking solutions offered by Wakoopa (https://www.wakoopa.com/), which is currently the leading company providing these services. This is also the solution used in our case study. Unlike found data sources (e.g., online platforms), which require selecting existing data sources or platforms (e.g., Twitter) and then extracting available traces (e.g., tweets), metered data are provided using samples of individuals who install tracking solutions on their devices. As for surveys, these samples can be built using both probability and non‐probability sampling approaches. As such, errors do not come from issues regarding how representative online platforms are, or the way in which to sample traces or users from those platforms, but rather from traditional sampling problems as well as the challenges introduced by asking participants to install tracking technologies on their devices.

### 2.2 Classification of error sources

**FIGURE 1:**
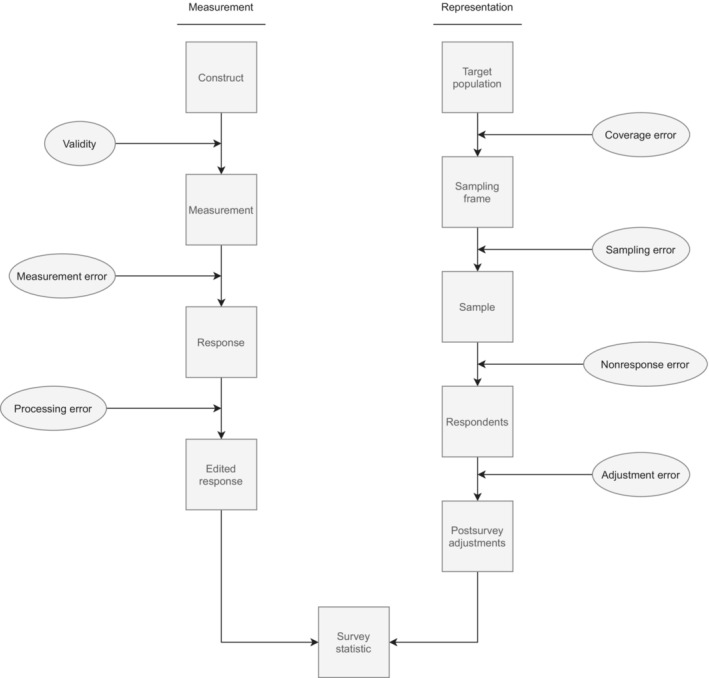
Reproduction of the total survey error framework by Groves et al. (2009)

Classifying error sources is a good way of thinking about data quality. Although data quality can be conceptualised in many ways (e.g., credibility, comparability, usability, relevance, accessibility), for the last 80 years, most error classification frameworks have explored those sources affecting data accuracy (Groves & Lyberg, 2010). Focusing on accuracy, Groves et al. (2010) built a highly influential error framework for cross‐sectional probability‐based surveys, the TSE, which links the steps of survey design, collection and estimation into the error sources and separates these into two different dimensions: representation and measurement (see Figure 1). Errors of representation refer to failures to measure eligible members of the population of interest. They include coverage, sampling, non‐response and adjustment errors. Errors of measurement refer to deviations between the concepts of interest for researchers and the processed measures collected, and include validity, measurement errors and processing errors. All these errors can affect the estimates' variance or bias, contributing to the overall mean square error of a statistic.

Although the TSE framework was initially conceived for probability‐based cross‐sectional surveys, in recent years, given the emergence of new types of digital data, researchers have expanded the TSE framework for some found data: Twitter (Hsieh & Murphy, 2017), online platforms (Sen et al., 2021) and Big Data in general (Amaya et al., 2020). These frameworks assume that the found digital traces, although presenting their own specific data collection processes and error sources, suffer from a number of representation and measurement errors that is comparable to surveys. Therefore, their errors can be identified and classified in a similar way to surveys. Despite these frameworks showing that the TSE framework can be expanded to digital trace data sources, their applicability to design‐based digital data sources such as metered data is limited, given the differences in data collection and sampling approaches exposed in Section 2.1. As such, this paper builds on this cumulative knowledge to propose a new framework for metered data.

## 3 BUILDING A TOTAL ERROR FRAMEWORK FOR METERED DATA

The TEM framework is designed to be flexible and applicable across different types of projects. To achieve this, first, we conceptualise the data collection and analysis process of metered data, and the error components, in a comparable way to surveys. Hence, we use the seven error components of the TSE presented by Groves et al. (2009) as a starting point (see Figure 1). Nonetheless, given the Big Data nature of metered data, we borrow the terminology used by Amaya et al. (2020) to refer to some error components, when it is better suited (i.e., we use ‘specification errors’ instead of ‘validity’ and ‘missing data errors’ instead of ‘non‐response errors’).

Moreover, although metered data are longitudinal by nature, most research has used it in a cross‐sectional way, aggregating data points to create a measure for a given period (e.g., the time spent visiting online news during a week). As for surveys, aspects of the metered data errors and their interaction might be different in longitudinal contexts. Considering that most past research has used metered data in a cross‐sectional way, and for the sake of simplicity, we developed the framework for cross‐sectional applications of metered data. Inspired by a previous adaptation of the TSE for longitudinal settings (Lynn & Lugtig, 2017), nonetheless, we highlight processes and error causes that could differ when researchers use metered data in a longitudinal way.

Finally, as for surveys, probability and non‐probability‐based sampling strategies can be used. Although substantial differences exist between both (Unangst et al., 2019), these are not meter‐specific and have already been discussed in previous research (Pew Research Center, 2016; Unangst et al., 2019). Thus, we present the data collection and analysis process when using a probabilistic approach and consider the error causes which would happen for a probability‐based approach. However, since most research to date has used metered online opt‐in panels, using the work by Unangst et al. (2019), we also highlight the steps and errors that are different or non‐existent in that case. It should be noted, nonetheless, that large variations can exist within online opt‐in panels (e.g., because of the methods used to recruit participants or select the samples).

## 4 THE TEM: METERED DATA FROM A PROCESS PERSPECTIVE

**FIGURE 2:**
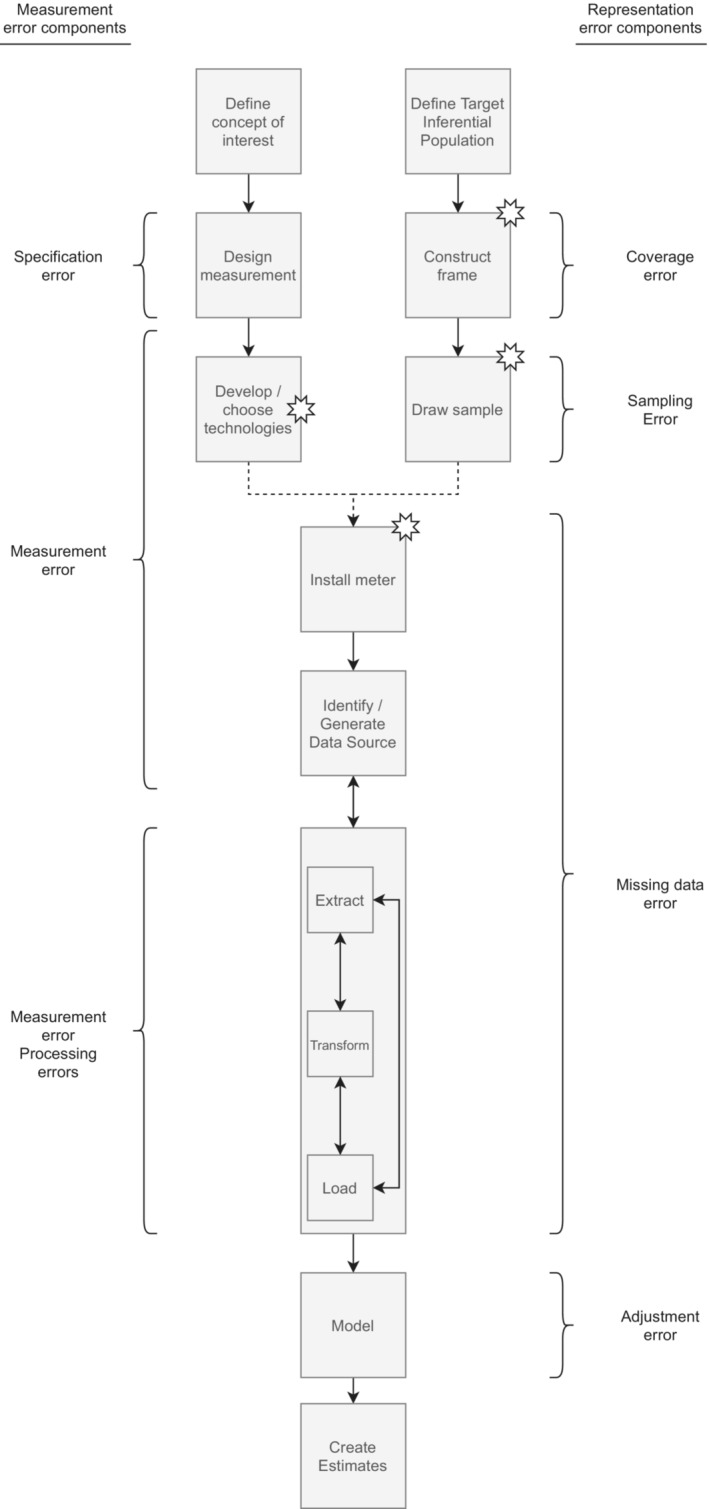
Data collection and analysis process for metered data. The stars indicate those processes that are different or non‐existent for opt‐in metered online panels.

Figure 2 presents an ideal workflow of the data collection and analysis process of metered data. For concision purposes, we focus on metered data only, not in combination with survey data.

Researchers conducting metered data research need to make decisions related to two main aspects: the sample and the measurement. On the measurement side (left set of boxes), the first decision is to *define the concept(s) of interest*. This means defining what the researchers want to measure (elements of information that researchers want to collect). To obtain data about these constructs, researchers need to subsequently *design the measurements*, that is, the specific instrument(s) to be used to gather information about the concept(s) of interest. In the case of surveys, measurements are survey questions (Groves et al., 2009). For metered data, measurements are the defined pieces of information from the participants' tracked online behaviour that are combined, and sometimes transformed, to compute a specific variable (e.g., all URLs that are considered as political articles).

Next, researchers need to *develop or choose the tracking technology* (or technologies) that will be used to obtain the information needed to create the measurement(s). SOM 1 in Data S1 provides a summary of the ones available through the most used provider of tracking solutions: Wakoopa. When using an opt‐in metered panel, participants have already installed some tracking technologies on at least one of their devices. Thus, researchers only have the possibility to choose the panel with the best‐suited technology (or technologies) for their project.

On the representation side (right set of boxes), the first step is to *Define the Target Inferential Population*, that is, who the researchers aim to draw conclusions about. The second step is to *Construct the Frame*. A frame is a list (e.g., emails of university students) or a procedure (e.g., a map of houses) that is intended to identify the elements of the target population. When using a metered panel, the panel acts as the frame (Unangst et al., 2019). Consequently, it acts as a list of individuals with a meter either installed or configured on at least one of their devices with an email associated. It does not aim to provide full coverage, but rather looks to include individuals with enough diversity to cover the panel's needs (Groves et al., 2009). The next step is to *Draw the Sample*, which means selecting a fraction of the frame from which measurement will be obtained. Ideally, this should be done using a probability‐based sampling approach. In practice, for metered panels, normally non‐probability sampling approaches are used, especially quota sampling (Ochoa & Paura, 2018).

Once the sample has been drawn and the technology chosen, sampled individuals can be asked to *Install the Meter* onto their devices. This usually involves asking participants to install or configure several tracking solutions across different devices (e.g., download an app onto an Android smartphone to track the behaviour on the device's browsers and download a plug‐in onto a Chrome browser on a Windows PC). This depends on (1) the traces needed by researchers and (2) the capabilities of the chosen technologies. Traces of interest are produced when participants connect to the Internet through specific web browsers and apps installed on a device that is connected to a specific network (i.e., home Wi‐Fi or 4G data plan). From now on, we will call these combinations of device/app/web browser/networks the *targets* to track. In some cases, more than one technology might be needed to track different targets on one device (e.g., a plug‐in for each web browser used, or a proxy for each network). Consequently, the process of inviting participants can be complex and may include various phases, with there being no standard way of doing it yet. Once correctly installed, the meter starts collecting data from the device and browser logs. When using an opt‐in metered panel, the process of installing the technology is beyond the researchers' control since researchers sample from a pool of already tracked individuals. Nonetheless, these individuals can still be asked to further install tracking solutions on top of the ones that they have, only for the specific project (Haim et al., 2021).

In the next step, the information collected by the meter is uploaded to a server that *Generates the Data Source*. Systems to collect and store data can be set in different ways depending on the technology. For example, for smartphone apps, Harari et al. (2016) propose to do the following: a portal server receives the data collected by the meter and checks them against the participant manager, which provides the unique user ID. The portal server subsequently stores the data collected in the data storage, which is normally a database that can handle large data sets (e.g., MySQL). These data sets allow for the data to be queried, extracted and, when necessary, allow for transformations to be applied for the construction of the final data set for the analyses. When using a metered panel, apart from the information generated after individuals have been sampled, the panel can also provide data already collected from when the participants joined the panel.

Once the data set of interest has been identified and/or generated, then comes the *Extraction, Transformation and Loading (ETL)* of the metered data. These steps follow a similar process as the one described by Amaya et al. (2020) for found data. Usually, they involve converting the raw and unstructured data into ‘structured’ variables. The steps can be done simultaneously or iteratively (e.g., extracting information, transforming it, loading it back and extracting it again). First, the process of *extracting* traces can involve (1) selecting subsets from the raw data set to perform further transformations or (2) extracting information and performing calculations to create ‘structured’ variables (e.g., counts of visits to specific URLs). After extracting traces from the whole data set, information might need to be further *transformed* to fit the defined measurement. Both simple transformations (e.g., from seconds to minutes) and complex codification procedures that may require using machine learning (ML) applications (see Grimmer et al., 2021, for a discussion of ML application for the social sciences) might be needed. For example, in order to create the variable ‘time spent visiting pro‐conservative news articles’, researchers need to code whether the content of the visited news articles can be considered as pro‐conservative or not. This can be done manually (but might be too time‐consuming) or through a supervised ML algorithm. Once this information is added to the extracted data set, researchers can create the desired variables. Finally, the extracted and transformed data sets are *loaded* and stored on the researchers' devices or servers. When using a metered panel, these steps might be done by the company or the researchers, depending on whether it is the latter who acquire the raw data set (i.e., information from all URLs visited by participants in addition to auxiliary information like timestamps) and perform the *ETL* steps by themselves; or if they buy a structured data set created by the fieldwork company following the researchers' guidelines (i.e., panellists in the rows, variables in the columns; variables based on the information from the raw data set).

Once a final data set is loaded, researchers can proceed to *Model*. This involves adjusting the data to better reflect the target inferential population. As such, it can include weighting for missing data, non‐response or coverage deficiencies and/or imputation for missing data. Finally, with the adjusted and modelled data, an estimate can be created (e.g., the mean hours of media consumption).

## 5 THE TEM: METERED DATA FROM A QUALITY PERSPECTIVE

Each step of the process from constructing the frame to creating the estimates contains some risk of errors. We consider that metered data are affected by the same error components as the ones presented in the TSE (Figure 1). They differ, however, in some of their characteristics and the causes behind them. In the following subsections, we conceptualise those error components for metered data and present their specific error sources.

Metered data and surveys share a similar process when it comes to drawing the sample from the frame, contacting sampled units and adjusting the estimates, and consequently, some error causes are similar or shared with surveys. Since those error causes have been explored extensively (Biemer, 2010; Groves et al., 2009), here we mainly discuss those specific to metered data. Table 1 summarises all meter‐specific error causes, by component.

**TABLE 1:** Specific error causes for metered data by error component

Error components	Specific error causes
Specification errors	‐ Defining what qualifies as valid information
	‐ Measuring concepts with by‐design missing data
	‐ Inferring attitudes and opinions from behaviours
Measurement errors	‐ Tracking undercoverage
	‐ Technology limitations
	‐ Technology errors
	‐ Hidden behaviours
	‐ Social desirability
	‐ Extraction errors
	‐ Misclassifying non‐observations
	‐ Shared devices
Processing errors	‐ Coding error
	‐ Aggregation at the domain level
	‐ Data anonymisation
Coverage errors	‐ Non‐trackable individuals
Sampling errors	‐ Same error causes as for surveys
Missing data error	‐ Non‐contact
	‐ Non‐consent
	‐ Tracking undercoverage
	‐ Technology limitations
	‐ Technology errors
	‐ Hidden behaviours
	‐ Social desirability
	‐ Extraction errors
	‐ Misclassifying non‐observations
Adjustment errors	‐ Same error causes as for surveys

### 5.1 Specification errors

A specification error (also known as [in]validity) arises when the concept being measured differs from the concept of interest (Biemer, 2010). For surveys, this arises when the question and scales do not properly measure the defined concept (e.g., the wording refers to something else). For metered data, errors might occur when the traces used to build the variables do not properly match the concept of interest. For instance, to construct the measurement ‘average time spent consuming political news’, the list of visits to URLs considered as ‘political news’ must be defined, and then the time spent on them must be added. If this defined measurement instrument deviates from the concept of interest, for instance by defining some non‐political content as political, specification errors appear.

#### 5.1.1 Defining what qualifies as valid information

For surveys, the words used in the request for an answer and the scale categories can affect how valid a measurement is (Saris & Gallhofer, 2014). Equally, when constructing a measurement for metered data, researchers must define which pieces of tracked information should be used and which not to measure behavioural or attitudinal concepts; for instance, whether to consider URLs as fake news or not (Guess et al., 2020). If, due to these specifications, the defined measurement instrument deviates from the concept of interest (e.g., including non‐fake news), specification errors are introduced.

#### 5.1.2 Measuring concepts with by‐design missing data

Researchers might decide to measure a concept even when they are aware that part of the data is missing by design. For instance, Guess et al. (2018) intended to measure the total fake news consumption of a sample of Americans during the 2016 presidential election. However, the authors collected data only from metered PCs. Thus, by design, the total fake news consumption could not be measured, but only the fake news consumption from the PCs. However, the authors used the collected data to make inferences about the total fake news consumption, making the (strong) assumption that total fake news consumption can be inferred from the fake news consumption found on PCs. If this is not the case, specification errors occur.

#### 5.1.3 Inferring attitudes and opinions from behaviours

Metered data collect behavioural information which can be used as a proxy to measure online behaviours. Other types of digital trace data have been used to measure attitudes and opinions. For instance, Barberá (2015) inferred individuals' left/right position based on the Twitter accounts they followed. If a behavioural indicator (e.g., URLs visited) is used to infer about attitudes or opinions (e.g., left/right position) without a solid theory behind it, it might produce weaker relationships, affecting the validity of the measurement.

### 5.2 Measurement errors/missing data errors

When using metered data, measurement and missing data errors can be confounded. As such, we discuss them together. For surveys, the measurement consists in (at least) one question. Participants can either answer or not answer (for whatever reason). Those not providing an answer are considered as missing. Since no information is available from them, they are excluded from the specific analyses. Missingness might happen at the unit level (i.e., no information is available for any measure for a given unit) or at the item level (i.e., information is not available for an item for a given unit). When data are missing, estimates are drawn on a subset of the sample. This can produce *missing data errors* if missing data differ systematically from the available data. For those answering, their answer might deviate from their true values (e.g., their self‐reported income does not match their real income), introducing *measurement errors*. This can happen, among other reasons, due to human memory limitations, interviewer influence, deliberate falsification or comprehension errors.

For metered data, a measurement is understood as the defined traces to use and how to transform them (see Section 5.1). Tracking solutions are used to collect these traces, which are then transformed into usable variables. This process can fail in at least two ways. First, undefined traces might be wrongly collected and/or classified as correct (e.g., behaviours done by third non‐tracked individuals). Thus, more traces are observed than those needed, provoking a measurement errors (e.g., for univariate analyses, it normally leads to a similar phenomenon as with over‐reporting). Second, defined traces might not be collected and/or wrongly excluded when creating the variables. This can lead to observing *part* or observing *none* of a participant's behaviour. On the one hand, if we observe part of the behaviour, the observed values are smaller than the participant's true behaviour, leading to measurement errors (e.g., similar to under‐reporting in surveys). On the other hand, not observing any of the defined traces leads to a lack of observation, which (as for surveys) should lead to the participants being excluded from the analyses (the lack of behaviour cannot be considered real, so the real value is unknown), introducing missing data errors. Nonetheless, given the nature of metered data, a lack of data (e.g., no adult website URLs recorded) might mean a true absence of behaviour (the individual has not visited any URL of interest) or a failure to capture data (e.g., the participant deactivated the meter to visit such URLs). Therefore, deciding whether the lack of information is considered as missing requires additional information and often depends on the researchers' judgement (see Section 5.2.9 for a more in‐depth discussion of the misclassification problem). This might not be the case, nonetheless, when measuring non‐behavioural concepts that require observations of specific behaviours. For instance, to compute the participants' left/right orientation using their visits to political news media websites as a proxy, for participants who do not visit any news media website, no left/right value can be computed.

In short, missing data and measurement errors might be confounded since the same sources of error can lead to each of them depending on (1) how many traces are missed, (2) the behavioural or non‐behavioural nature of the concept and (3) how researchers deal with the observed absence of behaviours.

#### 5.2.1 Non‐contacts

In order to collect metered data, sampled individuals need to be contacted and asked to install the meter. Regarding surveys, researchers might fail to contact some of the sampled units. For instance, mail or email invitations might never arrive or be seen by a sampled unit. In this scenario, the sampled individual does not become a participant, producing a missing data error. No measurement errors are introduced by non‐contacts.

#### 5.2.2 Non‐consents

Once contacted, individuals are asked to install the meter onto at least one of their targets. Some sampled units might not be willing to do so because of a variety of reasons. For instance, if the project requires long tracking periods, some participants might not be willing to participate in the study. Hence, no information is collected for those sampled units that do not become participants. Exploring the Netquest opt‐in metered panels in nine countries, Revilla et al. (2021) found that between 28.1% (United States) and 53% (Colombia) of those invited accepted to install the meter. No measurement errors are introduced by non‐consents.

#### 5.2.3 Tracking undercoverage

Although researchers are normally interested in measuring individuals' behaviours, meters measure the traces left by individuals when using specific targets. To measure the complete behaviour for a specific concept, meters must be installed on all specific targets used to engage in the given behaviour. Nonetheless, several reasons might prevent targets from being tracked:

1. *Non‐trackable targets*. Some of the targets used by participants might not be trackable with the chosen technology (or technologies). Hence, information cannot be collected from them. For instance, tracking apps cannot be installed onto iOS devices due to Apple's terms of service.
2. *Meter not installed*. Individuals who consent to being tracked must install or configure the meter into their targets. Even if participants agree to install the meter, they might finally decide not to do so (e.g., after reading the instructions they realise it is too much of a burden) or might fail to successfully do it in some targets (e.g., low IT skills, technical problems).
3. *Uninstalling the meter*. Participants who installed the meter at the beginning of the study can change their mind over time (e.g., lack of memory in the device, change on privacy concerns) and decide to uninstall the meter. Moreover, some participants may uninstall the meter accidentally. Both can happen for some or all the tracked targets. Data are unavailable from the moment the meter is uninstalled.
4. *New non‐tracked targets*. During the study, participants might purchase new devices or substitute old ones, switch to new browsers or start to use new networks. If these new targets are not tracked, their information is lost.

When any of these problems occur, not all participant's targets are tracked, leading to tracking undercoverage. The evidence so far suggests that 53% (Spain, Revilla et al., 2021) to 68% (United States, Pew Research Center, 2020) of metered participants do not have the meter installed on some devices. As a result, for some specific concepts of interest, part or all of the data from some participants might not be observed. When the loss of information is partial, this induces measurement errors (see Section 5.2). When it is absolute, researchers observe a lack of behaviour. In this scenario, and for behavioural concepts, whether missing data or measurement errors are introduced depends on the researchers' decisions (see section 5.2.9). For non‐behavioural measures (e.g., left/right position), an absolute loss of information provokes missing data errors.

For longitudinal research, if the level of undercoverage fluctuates over time, measures of change can be affected by the potential fluctuations in measurement error sizes.

#### 5.2.4 Technology limitations

Tracking technologies are subject to limitations. These prevent them from capturing some data types, in particular, currently: (1) not all available tracking technologies allow for behaviours happening in incognito mode to be captured. (2) Although most technologies can capture domain‐level information (i.e., theguardian.com) for all web page types, some approaches cannot capture sub‐domain‐level information (i.e., theguardian.com/sport/…) for https sites. (3) Behaviours happening inside apps (e.g., profiles visited within the Twitter app), to the best of our knowledge, cannot be captured with any current technology. (4) HTML content cannot be obtained from all tracking technologies. Therefore, depending on the technologies used, some information might not be trackable. Technology limitations can lead to both measurement and missing data errors depending on whether all traces are unobserved or not, the researchers' handling of the absence of behaviours, and the behavioural or non‐behavioural nature of the concepts. For longitudinal uses, if technology limitations vary over time (e.g., a new version solving some of the limitations or introducing new ones), measures of change can be affected by the variations in measurement error sizes.

#### 5.2.5 Technology errors

The meters, like any technology, can suffer from technological errors. If the meter stops recording or fails to correctly record information, information is lost. Several reasons can lead the technology behind the meter to fail: (1) the devices or third‐party apps might shut down the ability to collect data when devices are low on battery, to reduce the devices' energy consumption. (2) If the meter is working through a proxy, the proxy generates raw data that must be processed to identify which part of the tracked traffic was done passively by the device (e.g., downloading Facebook information) or actively by the participant. This is normally done by trained algorithms. However, this is not completely accurate. (3) Since tracking technologies are built on top of OSs and browsers when new versions of the software are released, they can prevent the technologies from working, causing a loss of information until the technology is adapted to the new version. These errors can provoke an incorrect collection or a loss of information.

Technology errors can lead to both missing data errors and measurement errors. In addition, for proxies, if there is an incorrect collection of information (e.g., the algorithm incorrectly categorises a passive behaviour done by the device as an active behaviour done by the participant), this produces a measurement error similar to over‐reporting. For longitudinal uses, technology errors have a similar impact as technology limitations.

#### 5.2.6 Hidden behaviours

Some technological approaches allow participants to disconnect the meter or to configure blacklists of domains not to be tracked. For instance, this can be used to avoid sharing information when dealing with online banking or visiting sensitive web pages.

Hidden behaviours can lead to both measurement and missing data errors depending on whether all traces are unobserved or not, researchers handling of the absence of behaviours, and the behavioural or non‐behavioural nature of the concepts. For longitudinal studies, measures of change can be affected by modifications of the blacklisted web pages or how the meter disconnection is allowed and/or used.

#### 5.2.7 Social desirability/Hawthorne effect

Participants might change their behaviours if they know that they are being observed (Jürgens et al., 2019). Consequently, their observed behaviours could deviate from their habitual (non‐observed) behaviours. This can especially affect sensitive behaviours, with participants behaving in a more socially desirable way when observed. Although no experimental research has been conducted yet, preliminary evidence using quasi‐experimental data suggests that individuals might not change their behaviour when observed (see Toth & Trifonova, 2021).

Changes in behaviours produce measurement errors unless they produce a complete loss of the information needed to compute a non‐behavioural measurement, in which case it should be considered as a missing data error. For longitudinal uses, if participants start behaving differently, measures of change can be biassed.

#### 5.2.8 Extraction/query errors

Often researchers do not extract all the data, but select specific domains, periods of time or individuals from which/whom to extract information. When specifying the domains or the time frame, incomplete or erroneous specifications can generate measurement errors. For instance, in the case of URLs, if a fake news domain is not specified in the query to extract data, this would underestimate the total fake news consumption. This is not a specification error since the error is produced not from the conceptualisation phase but rather as a mistake when creating and executing the queries used to extract the specified data. Extraction errors can lead to both missing data and measurement errors. Besides, problems with the query can leave sampled participants out of the final database if their information is not extracted.

#### 5.2.9 Misclassifying non‐observations

When extracting data from the entire data set to create metered data variables, only available tracked traces can be used. When no traces are observed for a specific defined measurement, the only reportable information is that no observation exists for that individual. For instance, a query can specify that a variable should be created reporting every time that the URL ‘theguardian.com’ has been observed during a determined time frame. If an individual does not present any observation containing this URL, the query can report that there is no observation. By default, this can be set to missing, or to 0. This lack of data can be due to a true absence of behaviour or a failure to capture data. In a perfect scenario, non‐observations should be treated differently depending on whether they are real or the result of some of the previously mentioned error sources (e.g., tracking undercoverage). Nonetheless, metered data alone do not provide enough information to make this decision. Therefore, researchers might misclassify an individual when deciding. If an individual is misclassified as presenting a lack of behaviour instead of being considered as missing, this inflates measurement errors and deflates missing data errors. Conversely, misclassifying a true lack of behaviour as an error‐induced one inflates missing data errors. Hence, misclassifications can introduce missing data or measurement errors, depending on their nature.

#### 5.2.10 Shared devices

Metered data are produced by individuals using specific devices. Devices, nonetheless, can be shared between different individuals. Revilla et al. (2017) found, for the Netquest opt‐in metered panel in Spain, that more than 60% of desktops, 40% of laptops and tablets and 9% of smartphones used to go online by the participants were shared to some degree. Let us assume that researchers want to measure partisan news consumption. Participant 1 shares a metered PC with their father. During the metered period, Participant 1 does not visit any news media website. However, Participant 1's father consumes an average of 1 h of liberal news media outlets from the shared PC. Participant 1 is considered to present a liberal consumption pattern, although they did not visit any news media website. Now, let us assume that Participant 1 does in fact visit 1 h of conservative media outlets. Then, Participant 1 is considered to engage with both conservative and liberal media outlets equally, not being polarised. However, their true behaviour would be exclusively conservative.

Shared devices, hence, introduce measurement errors but no missing data errors. For longitudinal uses, if the shared devices patterns vary across time, measures of change can be affected by variations in the sizes of measurement errors.

### 5.3 Processing errors

Processing errors can be introduced after the data have been collected and before the estimation process. These errors create deviations between the variables used for estimation and the observed ones. For survey data, processing errors can be produced during data entry, coding, editing, disclosure limitation and variable conversions or transformations (Amaya et al., 2020). In the case of metered data, tracked traces might need to be extracted and transformed in specific ways before building the variables, to fit the researchers' needs. Thus, the processed variables can differ from the desired measures.

#### 5.3.1 Coding/categorisation errors

Metered data can take an unstructured form like URLs, text, images or videos. Unstructured data often need to be processed and transformed to be useful. This process might involve coding or categorising the unstructured data into classes, labels, sentiment, etc. This can either be done before extracting the data (e.g., coding domains and sub‐domains as ‘political’, and then using queries to group their URLs in those defined categories), or after (e.g., extracting the raw data set, coding each URL in it, and then building the variables). Categorisation, as a process, is related but different to the one presented in Section 5.1.1. In this step, the definitions are used to classify the specific information, for instance with coders looking at each URL and judging by using the given definitions. This can be done manually (e.g., using MTurk coders, Peterson et al., 2018), using ad hoc ML algorithms (e.g., supervised ML to categorise the topic of news articles, Peterson & Damm, 2019), or using already available third‐party ML algorithms (e.g., Google's Vision AI, Bosch et al., 2019). Manual coding can prompt the same errors as for survey data, that is, that different individuals coding the same raw data have different judgments or that coders systematically misinterpret and misclassify some information. ML solutions might also present problems. Indeed, recent work has found that label errors are ubiquitous in the test sets of most of the popular benchmarks used in computer vision algorithms (Northcutt et al., 2021). Even if classification algorithms correctly classify the information, labels can still be biassed. For instance, for many commercially available systems, images of female US members of congress receive three times more annotation about their physical appearance than about their profession, something which is not observed for their male counterparts (Schwemmer et al., 2020). If these algorithms were to be used to understand the type of images that individuals are exposed to when reading news articles, results would be systematically different depending on the proportion of articles about female/male politicians consumed. For longitudinal analyses, changes to the underlying characteristics of the ground truth data or the ML algorithms used could affect the size of the errors and have an impact on the obtained measures of change.

#### 5.3.2 Data aggregation

In some cases, the final analyses cannot be carried out using the data at the URL level due to vendors, privacy regulations or researchers' decisions. Then, data are aggregated at the domain level (e.g., the domain for theguardian.com/uk/sport/ is theguardian.com). Hence, information is lost. On the one hand, this can lead to some concepts not being measurable. For instance, it is not possible to measure the time spent visiting the sports section of The Guardian if all the URLs with theguardian.com/uk/sport/ are converted into theguardian.com. On the other hand, some concepts might be measured less accurately. For instance, if interested in the total time spent visiting sports articles, the time spent on sports outlets (Eurosport) can be measured accurately but not the one spent on generalist outlets (e.g., theguardian.com). Thus, the final measure underestimates the total time spent visiting sports articles.

#### 5.3.3 Data anonymisation

Data can be anonymised, that is, all the pieces of information that could lead to identifying participants are obscured. This can be done manually or using ML algorithms. Both approaches, however, can cause errors. Thus, relevant information that was not intended to be hidden can be lost (Ochoa & Paura, 2018).

### 5.4 Coverage errors

Coverage errors occur when the sampling frame from which the sample is drawn differs from the target population, either because units are excluded, wrongly included or duplicated. If researchers use a metered panel, coverage errors occur when the full panel differs from the target population (Groves et al., 2009). Although unquantifiable per se when using a metered panel, errors are linked to one or more panel practices: for instance, their refreshment strategies or if they blend samples from different sources. Researchers can qualitatively assess panels beforehand to potentially reduce these errors (Unangst et al., 2019).

#### 5.4.1 Non‐trackable individuals

Individuals might only use non‐trackable targets to access the Internet. Although these individuals might appear in the sampling frame, once contacted, they do not have the possibility of participating. Specific coverage errors related to trackable devices can often not be assessed until sampled units are contacted and the use of trackable devices is assessed. This type of coverage error can be solved if the sampled units using the Internet with non‐trackable devices are provided with trackable devices.

### 5.5 Sampling errors

Sampling errors arise due to the analysis of a subset rather than the entire population of interest. The causes behind sampling errors do not differ between survey and metered data. When units in the sampling frame have a zero chance of selection, these units are excluded from every potential sample drawn. If the excluded units differ from the non‐excluded ones in the frame, a bias is introduced. Sampling also introduces variance into estimates. For a given sampling design, many different samples can be drawn. Each sample, by chance, produces different values for the statistics of interest (e.g., average time spent visiting online political media outlets). Several factors can increase sampling variance, such as small sample sizes or the use of clustering.

When using an opt‐in metered panel, non‐probability sampling is used. Therefore, units are included with unknown probabilities and the sampling error size is unknown.

### 5.6 Adjustment errors

When modelling and creating estimates, researchers can make use of weighting or imputation strategies with the objective of improving the representativeness of statistical estimates. Since metered data can be based on probability and non‐probability samples drawn in a similar fashion as done for surveys, similar weighting and imputation strategies can be used, with similar risks of producing errors. Hence, deficiencies in missing data and coverage error weighting adjustments, as well as imputation for an item missing data, can introduce adjustment errors (see Mercer et al., 2017, pp. 256‐257 for an example).

In recent years, ML approaches have been considered to improve weighting and imputation adjustments, with some promising results (Dagdoug et al., 2021). The volume and richness of metered data might allow adjusting strategies to be more accurate when dealing with missing data. Nonetheless, it is still too early to know whether we should expect different sources of error when applying these approaches.

## 6 CASE STUDY: APPLYING THE TEM TO THE TRI‐POL PROJECT

The objective of the TRI‐POL project is to understand whether and how online behaviours are related to affective polarisation across Southern European and Latin American countries (https://www.upf.edu/web/tri‐pol). To this end, a three‐wave survey was conducted in Argentina, Chile, Italy, Portugal and Spain between September 2021 and March 2022, and matched at the individual level with metered data. The TRI‐POL web tracking strategy was designed using the TEM, to maximise the quality of its data.

Data were collected through the opt‐in metered panels of Netquest (https://www.netquest.com) in the five countries of interest. Cross quotas for age, gender, educational level and region were used in each country to guarantee a sample similar to these variables to the general Internet population. Survey questions were used to measure the participants' affective polarisation and other attitudinal and demographic variables, while metered data were used to measure variables related to the participants' general Internet use as well as their consumption of news media outlets, political news and social media.

TRI‐POL represents a good case study for four main reasons: (1) it focuses on the most frequently measured concepts so far using metered data (social media and news media consumption). (2) As with most past research, it uses a metered panel, as well as the most common tracking solutions provider (Wakoopa). (3) Its cross‐national nature allows for the testing of how the TEM framework can be used to create comparable cross‐national measures, which is key when comparing standardised relationships across nations (Bosch & Revilla, 2021). (4) Once completed, TRI‐POL data will be available with open access (check here once available: Torcal et al., 2022a, 2022b). The TEM allows for the transparent documentation and communication of the development of the data set, its limits and best practice when using it.

Below, we describe how the TEM was used to minimise the size of the error sources and/or quantify them. For concision purposes, we limit our consideration to the error sources that could realistically cause bias in our estimates of interest and be measured and/or improved within the TRI‐POL context. For those error sources, we also present empirical evidence about their prevalence and/or potential to introduce bias within the TRI‐POL datasets of Italy, Portugal and Spain. SOM 3 to 8 in Data S1 give more in‐depth explanations of the data used and the analyses performed to reach those results. A discussion of the other error sources is provided in SOM 2 in Data S1.

### 6.1 Specification errors in TRI‐POL

#### 6.1.1 Defining what qualifies as valid information

The TRI‐POL project aimed to measure more than 5000 concepts across five countries. Due to this big volume, it was key to develop a standardised strategy to create valid measurement instruments across topics and countries. Below, we briefly summarise our strategies to minimise and quantify the specification errors. A more in‐depth discussion is proposed in Bosch and Revilla (2022b).

**Strategy to minimise errors:** To operationalise each concept of interest into valid metered data measurements, we developed the following three‐step procedure:

1. *Definition of the lists of URLs/apps to be used*. Regardless of the concept (e.g., time in specific sites, type of content exposed), traces of interest are created when an individual accesses to a specific URL or app. Hence, we created comprehensive lists of URLs/apps where traces of interest would be produced for each concept. For instance, for the concept ‘online news media exposure’, we defined a list of all the URLs/apps considered as ‘news media articles’. This involved defining: (1) the news media outlets to be considered in each country and (2) the URLs to consider within each outlet.
2. *Definition of what a visit of interest is*. Even if an individual visited one of the defined URLs/apps, the generated traces could still not be relevant to the concept of interest. For instance, if what was being measured was whether a person read an article or not, only visits complying with the requirements for being considered as read should be used (e.g., a threshold of 120 s).
3. *Establishment of the time frame of interest*. Most of the TRI‐POL concepts of interest involved measuring average behaviours. To this end, the total sum of behaviours of an individual during the tracked period was divided by a given number of days. This had to be grounded in theory because the chosen time frame could affect the likelihood and prevalence of outliers and the skewness of the data, which can ultimately impact the estimates.

Following these steps not only helped to properly define the traces to use for each concept of interest but also highlighted the design choices for which not enough evidence was available to make informed decisions.

**Strategy to quantify errors:** When different design choices could be used (e.g., using different lists of the main news media domains in a country), and not a particular one was identified as being better, we computed one variable for each potential design choice. For instance, for the concept ‘online news media exposure’, we had to make different decisions, such as which list of news media domains to consider or how many days of tracking to use when computing the variables (see SOM 4 in Data S1 for the complete list of design choices that we considered). Nonetheless, given the available literature, we thought not possible to make an informed decision about which design choices to use (e.g., is it preferable to use 2 weeks of tracking or 1 week?). Thus, we created a variable for each potential combination, which resulted in 3573 variables to measure the concept of ‘online news media exposure’.

Mirroring what is normally done in the survey literature (see e.g., Smith et al., 2020), for those concepts for which we created more than one variable, we then computed the convergent, discriminant and predictive validity. Following the example of ‘online news media exposure’, in terms of convergent validity, we computed one correlation for each potential pair of variables. Therefore, we obtained 6,349,266 unique Pearson's correlation coefficients for each country. On average we found that, across countries, the correlation between the different computed variables was between .40 (Spain) and .51 (Italy), which can be considered as a sign of medium to low convergent validity. This indicates that, depending on the design choices made, variables could not be considered to measure the same latent concept of ‘online news media exposure’.

Finally, to make sense of this abundant amount of data, we applied random forests of regression trees to estimate the influence of the different design choices on the validity of measurements, drawing inspiration from the Survey Quality Predictor software (Saris et al., 2011). This informed us about (1) which measures to use in the final analyses and (2) the robustness of TRI‐POL results. (see SOM 4 in Data S1 for an in‐depth explanation about this). For example, for ‘online news media exposure’, we used this approach to investigate which design choices helped maximise the predictive validity of the measurements. We found that: (1) tracking participants in both PCs and mobile devices should be preferred over using only PCs or mobile devices (this contrasts with what most past literature has done); (2) when deciding which news media outlets to track, the top 50 most visited news media outlets of a country should be tracked, with little additional predictive power gained with extra tracked outlets; and (3) 10–15 days of tracking data should be used, with longer tracking periods not necessarily performing better. A more in‐depth discussion of these results can be found in Bosch & Revilla (2022a).

### 6.2 Missing data/measurement errors in TRI‐POL

#### 6.2.1 Tracking undercoverage

For TRI‐POL, we used a panel of self‐selected individuals who were already being tracked with specific tracking solutions. Undercoverage was expected (Revilla et al., 2017). However, asking panellists to install new tracking technologies was not possible due to time and budget constraints. Therefore, we assessed the prevalence of undercoverage and estimated the bias it introduced, as summarised below. For a more in‐depth explanation, we refer to Bosch and Revilla (2022b).

**Strategy to quantify errors**: To assess the prevalence of undercoverage, two pieces of information were needed for each participant; which targets were tracked, and which targets they used to go online. The first piece of information was obtained using paradata and the second by asking participants questions about which devices and browsers they used to access the internet during the 15 days before the first survey wave (see SOM 5 in Data S1 for the exact formulation used). Combining both sources of information, we estimated the proportion of undercovered individuals, as well as the number and types of non‐tracked devices and browsers. Across the different countries, we found that the between 80.5% (Spain) and 85.7% (Portugal) of TRI‐POL participants had at least one device or browser not tracked. Hence, tracking undercoverage is highly prevalent in the TRI‐POL data sets.

The combination of survey and tracking paradata also allowed us to develop an approach to estimate the bias introduced by undercoverage. Specifically, using metered data from the sub‐sample of fully covered individuals, we simulated how different levels (% of participants not having all PCs or mobile devices covered) of undercoverage would cause a bias for a set of univariate and multivariate estimates from their true values (see SOM 5 in Data S1 for more detailed information about this approach). As an example, we simulated the bias that device undercoverage could introduce to the results obtained for the measure ‘average time spent on the Internet’, which represents the average time spent on any URL or app across all tracked targets, for the 15 days prior to the survey being answered. We estimated that, when using the TRI‐POL data set, tracking undercoverage could underestimate the average time spent by participants on the Internet by 3.9% (Spain, in a scenario with 25% of the sample without information about PC behaviours) to 25.8% (Spain, 75% of the sample without information about mobile behaviours). This would imply that, at the actual levels of participants in the TRI‐POL samples with all their PCs or mobile devices not tracked, the observed average time spent on the Internet might be underestimated by around 14%, across the different countries. This estimate does not take into account the effect of individuals having some but not all of their PCs or mobile devices not tracked, hence, the real bias is most likely even higher. A more in‐depth discussion of these results can be found in Bosch (2022).

#### 6.2.2 Technology limitations

To reduce errors produced by technological limitations, ideally, we would: (1) identify the digital traces and their characteristics and (2) design or select the technologies allowing for their collection with the highest level of accuracy possible. In the TRI‐POL project, the control over (2) was limited since data were collected through an already existing metered panel. Nonetheless, we defined strategies to quantify the prevalence and potential impact of these limitations on the estimates.

**Strategy to quantify errors**: We asked Netquest to provide information on all their tracking solutions and their limitations (what devices and traces they could or could not track, their level of accuracy, potential limits and interactions between devices/OSs and technologies; see SOM 1 and 6 in Data S1). We then combined this information with the paradata about the technologies used to track each participant to compute the proportion of participants who could be affected by specific technology limitations (e.g., not being able to track incognito tabs). This allowed us to compute of proportion of participants:

1. *Not trackable in incognito mode*: we found 13.5%, 6.2% and 8.1% of participants affected by this technological limitation in Italy, Portugal and Spain, respectively.
2. *Without sub‐domain information*: those represented 20.5% (Italy), 12.0% (Portugal) and 14.8% (Spain) of participants.
3. *Without in‐app information*: given that the tracking solutions used by Netquest cannot capture in‐app behaviour, 100% of TRI‐POL participants were affected by this, across all countries.

The nature of these limitations, nonetheless, prevented us from assessing the extent to which they might influence the final estimates. For instance, since the information from in‐app behaviours cannot be obtained with any current approach, it is not possible to assess how much and which type of information is missed.

#### 6.2.3 Technology errors

Although researchers have little control over technology errors when using a metered panel, we designed the following strategies.

**Strategy to minimise errors**: First, we identified whether the tracking solutions used were susceptible to being shut down by energy‐saving apps and/or built‐in features of the devices. This was not the case. Second, we limited the sampling pool to participants with up‐to‐date meters, which are better equipped to deal with the latest OS versions. Third, due to some of the errors, meters can stop tracking entirely. As such, sampled participants might not produce any metered data during the tracking period. To avoid this, we excluded participants without any tracked behaviour during the last month from the sampling pool. This could exclude very low‐frequency internet users, but their very presence should be rare in an opt‐in online panel. Finally, manually configured proxies sometimes produce inaccurate results. An approach to avoid these problems could be excluding participants using iOS devices. We considered that the undercoverage errors introduced by this would outweigh its benefits. As such, TRI‐POL data from iOS devices might potentially be affected by measurement errors, which should be quantified.

**Strategy to quantify errors**: The nature of proxy errors makes them hard to quantify since it is complex to understand when and how the classification algorithm might have failed deciding which traces to include or exclude. However, indirect strategies can be used to test whether iOS users present different measurement properties. As an example, we tested whether being tracked on an iOS was associated with the absolute difference between the self‐reports and metered data for the variable ‘average time spent on the Internet’ (Absolute difference = | *Self‐reported time – Tracked time* |, see Araujo et al., 2017). Although we expect both the survey and metered measures to be affected by errors, a significant effect of being tracked on an iOS could indicate that participants tracked on an iOS present different measurement properties. SOM 7 discusses in more depth the exact analyses conducted. We found that being tracked on an iOS significantly increases the absolute difference in Italy and Spain, but not in Portugal. Specifically, in the TRI‐POL data set, being tracked on an iOS device is associated with having and absolute difference 56.8 and 57.6 min larger than for those not tracked on an iOS. Therefore, controlling for different potential confounders, we see that the mismatch between the survey and the metered data measures for iOS respondents is substantially higher than for those not tracked on an iOS. This could indicate that measures coming from iOS devices present different measurement properties, potentially of lower quality.

#### 6.2.4 Hidden behaviours

Participants had two potential ways of hiding their behaviours: blacklisting domains and disconnecting their tracking technologies.

**Strategy to minimise errors**: We asked Netquest for their blacklisted domains in order to know whether some behaviours would be missing by design. None of TRI‐POL's defined URLs were blacklisted.

**Strategy to quantify errors**: No information was available about whether participants disconnected their trackers or not, nor was there information about the types of content triggering this. Besides, considering that participants had the tracking technologies installed before sampling them, we could not apply quasi‐experimental approaches like the ones proposed by Toth and Trifonova (2021). Consequently, we could not quantify (1) whether participants disconnected their meters and (2) the extent to which this could cause a bias in TRI‐POL's estimates.

#### 6.2.5 Misclassifying non‐observations

Minimising and quantifying misclassifications required collecting as much information as possible about the errors which could provoke error‐induced non‐observations, to clearly differentiate which non‐observations should be considered real and which not.

**Strategy to minimise errors**: Information about other error sources was used to decide which non‐observations were coded as real (0), as error‐induced (NA) or as unclear (specific code). For instance, for a few concepts, we directly asked participants whether they had visited specific domains with non‐tracked targets (see SOM 7 in Data S1 to check the questions asked). This information allowed us to discern between real and undercoverage‐induced non‐observations, potentially reducing the extent to which misclassification could affect the estimates.

**Strategy to quantify errors**: This same information also allowed us to compute the prevalence of undercoverage‐induced true non‐observations across different topics, individuals and countries. For instance, we computed the proportion of individuals with undercoverage‐induced non‐observations for Facebook, Twitter and the five most popular news media outlets in each country (see SOM 8 in Data S1 for a more in‐depth explanation). Across the different countries and domains, we found between 3.1% (Italy, GazzetaSud) and 25.9% (Spain, RTVE) of participants with undercoverage‐induced non‐observations. These proportions, nonetheless, varied highly depending on the domain of interest. In terms of social media, Facebook presents a low proportion of participants with undercoverage‐induced non‐observations (from 6.7% in Portugal to 8.4% in Spain), whereas the proportion of affected participants is substantially higher for Twitter (11.7% in Spain and 19.0% in Italy). The proportion of individuals with undercoverage‐induced non‐observations for news media outlets was on average between 10.1% (Italy) and 15.1% (Portugal). Thus, in the TRI‐POL data set, there is a non‐negligible risk of increasing the size of the estimate's measurement errors if these participants are not excluded from the analyses.

In order to quantify the extent to which misclassifying these non‐observations as true lacks of behaviours could introduce bias, one could apply a similar simulation strategy as for undercoverage (see Section 6.2.1): undercoverage scenarios can be combined with misclassification scenarios; simulated non‐observations should be randomly misclassified to approximate the effect that this could have in the full data set.

### 6.3 Processing errors in TRI‐POL

#### 6.3.1 Coding/categorisation errors

The TRI‐POL project measured variables about the participants' consumption of specific news media content (e.g., political, national, opinion). Thus, we had to define what we considered, for instance, to be ‘political’ or ‘opinion’ content. Using these definitions, we created guidelines to code subdomains of the tracked news media outlets as ‘political’ or not, ‘opinion’ or not, etc. (see https://www.upf.edu/web/tri‐pol/documentation‐and‐data‐archive). Using the guidelines, human coders went through all the listed news media outlets in each country and categorised their sub‐domains as mostly containing national, international, regional, opinion or other articles (e.g., theguardian.com/politics mostly lists URLs covering political news and, as such, it is considered as political).

**Strategy to minimise errors**: Coders were required to have language and country‐specific knowledge. To supervise that the coding approach was applied in a comparable way across countries and to spot potential coding errors, another researcher supervised all the coders' work.

**Strategy to quantify errors**: Given the time and budget constraints, only one coder per country was used for the full data set, preventing us from computing inter‐coder reliability. Therefore, it was not possible to quantify how codes varied across coders.

## 7 DISCUSSION

Metered data are increasingly being used to understand people's online behaviours. In this article, we propose a Total Error framework for Metered data, the TEM, which documents and conceptualises the data generation and analysis process of metered data. It also distinguishes the various kinds of errors that might arise during each step in the process. By expanding the TSE for metered data, the TEM framework can be used by researchers from different backgrounds, to improve documenting, quantifying and, when possible, minimising errors. Given the framework's flexibility, the TEM can be applied to stand‐alone metered data projects and projects combining metered data with surveys, to probability and non‐probability‐based sampling approaches and to cross‐sectional and longitudinal research.

### 7.1 Limitations and future research

The TEM framework presents some limitations. First, as the TSE, the TEM only considers a definition of data quality. Other factors should be considered when deciding whether to use metered data (e.g., cost, timeliness, risks). In particular, privacy and ethical issues must be considered when planning to collect metered data. Truly informed consent might be more difficult to obtain than in surveys due to the limited understanding of some participants regarding the data being collected (Revilla, 2022). Moreover, metered data should not be shared nor made publicly available in its raw form if it can represent any risk to the participants' privacy, even if this could make some results not fully reproducible.

Second, the TEM considers the errors of the metered data independently. Nonetheless, metered data have mostly been collected in parallel with surveys. In such cases, one must consider the potential trade‐offs between the active (surveys) and passive (metered) data collection parts of their projects. These trade‐offs might vary across the many potential ways in which surveys and metered data can be combined (Revilla, 2022). In‐the‐moment surveys represent a good example (Revilla, 2022). By tracking what people do online with metered data, in‐the‐moment surveys can be triggered when a participant shows a specific behaviour, allowing (1) for the measurement of new survey concepts and (2) the enhancement of metered data (for instance, by recording the reasons behind behaviours). Nonetheless, sending surveys after a specific behaviour might make participation in the project more burdensome, as well as the monitoring of their behaviours more evident. This could potentially increase social desirability and the likelihood of dropping out even if the (limited) existing evidence does not seem to support these ideas (Ochoa & Revilla, 2022a, 2022b). Although our framework does not directly address the particularities of these projects, its similarity with the TSE allows researchers to use both frameworks simultaneously to consider potential interactions. Nonetheless, further research is needed to get a better understanding of how to theoretically and empirically do this.

Third, although this paper discusses and exemplifies the strategies followed in the TRI‐POL project to quantify error sources, standardised approaches to quantify metered data errors still need to be developed and tested. The TEM framework, however, can serve as the foundation for future empirical research. Based on our experience, future research should explore at least three areas: (1) since the representation and measurement processes of metered data resemble those of surveys, approaches used to quantify surveys errors could, in principle, be adapted to metered data (e.g., GMTMM for administrative and survey data, Oberski et al., 2017). (2) Combining metered data with survey data can help have a better understanding of the quality of both sources. (3) Metered data allow for the creation, for each concept of interest, of dozens or hundreds of variables by simply altering the specifications of the queries. This might allow for ML algorithms to be fed with hundreds or thousands of quality estimates, for a single study, to predict what characteristics might yield the least biassed estimates.

Fourth, even if this paper presents some empirical evidence regarding the data quality of the TRI‐POL datasets, the prime goal of these examples is to showcase how the TEM might help when designing instruments based on metered data, and how the quality of these can be empirically tested. More in‐depth research must be conducted to get a good understanding of (a) the best approaches to measure metered data quality, (b) the general quality of metered data and (c) the relevance of each error source.

Fifth, in the coming years, ML might play an important role in future uses of metered data. For instance, ML might increasingly be used to process metered data and/or the use of metered data in predictive statistics might become more prevalent (Hofman et al., 2021). If this happens, the TEM framework can be used to accommodate current debates on the challenges of ML for big data sources, and potential solutions (see Qiu et al., 2016 for a good summary of this). This could mean understanding how ML might amplify (Wang & Russakovsky, 2021) or reduce (Ramirez et al., 2019) already existing biases or how and when automatisation errors could introduce new sources of error.

Finally, although key differences separate metered data from other digital trace data sources, the TEM framework can be applied to better understand the errors of other digital trace data sources or used as the foundation for new frameworks. On the one hand, metered data are similar to platform trace data or data donations; error sources like defining what qualifies as valid information or tracking undercoverage can be translated. On the other hand, parallels can be drawn with traces like GPS or call log data. In these cases, most measurement, processing, missing data and coverage errors might apply (with potential differences in the details), since these have to do with the technologies being installed and properly working.

### 7.2 Conclusions and practical recommendations

Well‐designed metered data can be a very useful resource. By developing the TEM, our goal was twofold: to help researchers (1) understand the limitations of already published research using metered data, and (2) to design metered data collection strategies that minimise the error size and allow for the quantification of the remaining errors.

Most research done to date has not discussed the potential limitations of metered data in enough detail. For instance, it is common not to report the tracking solutions used, nor how measurements have been defined, nor the prevalence of errors such as tracking undercoverage. This is not an adequate practice since it does not provide enough information to judge data quality, and it is not in line with current good practices in survey research (e.g., comparable to not reporting response rates). This is even more pressing if we consider that, when applying the TEM to quantify the error sources affecting the TRI‐POL data sets, we have found that some errors such as tracking undercoverage, invalidity, technology errors or the misclassification of non‐observations are highly prevalent. Moreover, we showed that invalidity and tracking undercoverage have the potential to bias the results obtained (e.g., underestimating univariate estimates or reducing the predictive power of variables in multivariate models).

Considering this, and based on the TEM and our experience with TRI‐POL, we propose some practical recommendations when using metered data:

1. Clearly define the list of traces (and how to transform them) to create valid measurement instruments. If it is not possible to make an informed decision and differences in the validity of different design choices are expected, create several measurements and test the robustness of the results and/or validity.
2. Consider the potential consequences that the different technologies can have on data quality before deciding which one(s) to use. If this is out of control, list all their limitations and report their prevalence and how these might affect the final estimates.
3. Clearly define what targets need to be tracked and try to maximise their coverage. If this is not possible or is out of your control, collect auxiliary information to assess its prevalence and potential to introduce bias.
4. Be mindful that tracked devices might be used by non‐participants. Try to develop strategies to minimise or assess how this can affect the estimates.
5. Non‐observations might be caused by errors. Auxiliary information should be collected to classify non‐observations as real or induced by errors, in order to deal with them accordingly.
6. Develop strategies to minimise and correct for human or machine induced errors when extracting and transforming the raw data into observed variables. Metered data projects can quickly become complex, involving many steps that might be sensible to errors.

To conclude, collecting high‐quality metered data is complex and involves a high degree of uncertainty. Decisions often require balancing many pros and cons with limited information. This does not imply that metered data should not be used, or that previous research might be biassed. It means, instead, that working with metered data requires a high degree of care and transparency and that further research is needed to help researchers to optimise their use of such data.

## Supporting information




**Data S1**. Supporting information

## Data Availability

The data that support the findings of this study will be openly available in OSF at https://osf.io/3t7jz/ (DOI: 10.17605/OSF.IO/3T7JZ)
