# Supplementary material for: When survey science met web tracking: Presenting an error framework for metered data
Source: J R Stat Soc Ser A Stat Soc. 2022 Nov 6;185(Suppl 2):S408–36. doi: 10.1111/rssa.12956 (PMC10100245; doi:10.1111/rssa.12956)
Supplement: Supplementary file 1 — Data S1. Supporting information [file RSSA-185-S408-s001.docx]

**Supplementary Online Material (SOM)**

**SOM 1 – Tracking solutions offered by Wakoopa, and their limitations**

Table 1 shows the different technologies that the company Wakoopa (currently the main provider of tracking solutions, and the one used by Netquest) uses, as well as the type of information that these collect and for which devices they are used.

**Table 1. Data collectable by tracking technology and target, for Wakoopa**

|  |  | **PC app** | **PC plug-ins** | | | **Android SDK** | **iOS proxy** |
| --- | --- | --- | --- | --- | --- | --- | --- |
|  |  |  | **Chrome** | **Firefox** | **Safari** |  |  |
| **Online tracking** | |  |  |  |  |  |  |
| URLs | Http traffic | Yes | Yes | Yes | Yes | Yes | Yes |
|  | Https traffic | No | Yes | Yes | Yes | Yes | No |
|  | Incognito sessions | No | Yes | Yes | Yes | Yes | No |
|  | HTML | No | Yes | Yes | Yes | No | No |
|  | Time stamps | Yes | Yes | Yes | Yes | Yes | Yes |
| Apps | App name | - | - | - | - | Yes | Yes |
|  | App usage start time | - | - | - | - | Yes | Yes |
|  | App usage duration | - | - | - | - | Yes | Estimated |
|  | Offline apps | - | - | - | - | Yes | No |
|  | In-app behaviour | - | - | - | - | No | No |
| Search terms | Search terms | Yes | Yes | Yes | Yes | Yes | No |
| **Device information** | |  |  |  |  |  |  |
| Device type | E.g. desktop | Yes | Yes | Yes | Yes | Yes | Yes |
| Device brand | E.g. Xiaomi |  | No | No | No | Yes | Yes |
| Device model | E.g. S9 | No | No | No | No | Yes | Yes |
| Operating system | E.g. iOS | Yes | Yes | Yes | Yes | Yes | Yes |
| OS version | E.g. 10.1.2 | No | No | No | No | Yes | Yes |
| Internet provider | E.g. Voxi | No | No | No | No | Yes | Yes |

**SOM 2 – A discussion of why we could not minimize or quantify some error sources for TRI-POL**

This SOM briefly discusses why, in the TRI-POL project, we could not design strategies to minimize or quantify some of the error sources affecting metered data not mentioned in the main manuscript's text.

**Noncontacts and Non-consent:** This error source cannot be measured nor prevented for samples obtained through metered online panels since the invitation to install a meter is outside researchers’ control.

**Social desirability:** Currently, the best approach to assess the potential effect of social desirability on people’s behaviour is to use or combine tracking approaches that allow to collect traces from before participants installed the tracking solutions and compare these with post-installation traces. This should allow assessing whether installing the meter changed their behaviours. Unfortunately, this cannot be done with a metered panel, since sampled participants installed their technologies before being sampled.

**Shared devices:** Several approaches can potentially reduce the effects of shared devices and/or allow to assess the associated errors. First, depending on whether a fresh sample is used or not, participants can be asked to only install tracking solutions on non-shared devices, or we can only sample participants who use non-shared devices. This, nonetheless, could potentially introduce tracking undercoverage and/or selection errors. A more appropriate strategy would be to use algorithms to differentiate between the participant’s behaviour and third-person’s behaviours (Ochoa et al., 2017). This approach helps estimate the bias introduced by shared devices and select only participant’s traces to create the desired variables. Nonetheless, we could not apply this strategy since these classification algorithms are trained using the raw data of participants, which we did not have access to. Hence, we expect TRI-POL data to be affected by shared-device-related measurement errors but do not know to what extent.

**Data aggregation:** Although we did not have access to the raw dataset, we were able to use all the tracking information to compute our desired variables. Hence, we do not expect data aggregation errors.

**Data anonymization:** Since we did not have access to the raw data, but to a structured dataset composed of pre-defined variables, there was no need to anonymize pieces of information from the raw dataset. Hence, we do not expect data anonymization errors.

**Non-trackable individuals:** Opt-in online panels are affected by coverage errors, but they are unmeasurable per se (Unangst et al., 2019). The problem of non-trackable individuals might make this problem worse. Given that we used a metered opt-in online panel, the prevalence and added potential bias of non-tracked individuals was not measurable.

**SOM 3 – Information about the TRI-POL data**

For the empirical analyses, we used web survey answers, metered data and paradata from the first wave of the TRI-POL panel dataset. Specifically, we focus on the data collected in Italy, Portugal and Spain. Data was collected through the metered panels of Netquest ([www.netquest.com](http://www.netquest.com)). The Netquest metered panels provide a pool of individuals with the meter installed, who can also be contacted to conduct surveys and, hence, link their online behaviour with their survey answers. When the panellists agree to join the metered panel, they must install the meter on at least one device (PC, tablet, or smartphone) and start sending information (passively) to Netquest to become part of the metered panel.

Cross quotas for age, gender, educational level and region were used to guarantee that the samples were similar on these variables to the general Internet population between 18 and 70 years, for each country. Data collection for the three waves took place between September 2021 and April 2022. In total, for the first wave, 3,548 respondents completed the survey until the end, 1,289 in Spain, 1,028 in Portugal and 1,231 in Italy. Challenges were faced when filling some of the specific cross-quotas with participants from the metered panel. This required supplementing in some cases with non-metered panellists. For the first wave, 993, 818 and 842 participants in Spain, Portugal and Italy respectively had the meter installed in at least one mobile or PC device. We will focus on this subsample of metered individuals.

Metered data was collected for the 15 days prior and posterior of participants starting each survey wave. The meter captured each URL (or app for mobile devices) accessed by the panellists, with timestamps for when the panellists first visited the URL, and the number of seconds in which the URL remained active in the browser. Participants were tracked on iOS and Android mobile devices, and Windows and MAC computers. Besides, we collected paradata from participants. For all individuals we were able to identify the technology with which they were being tracked, the type of device, the OS, whether it was a tablet or smartphone and, for plug-ins, the browser in which they were installed. Finally, the questionnaire focused on measuring, among others, political trust, participation and polarization, as well as several sociodemographic variables

**SOM 4 – Quantifying the validity of metered data measures**

As an example, SOM 4 briefly discusses the approach followed to quantify the validity of “online news media exposure.”

**Measuring online news media exposure**

Our first step was defining how to create the measurement of “online news media exposure.” The goal was to get a measure of participants average exposure during the project’s period of interest. Hence, we considered all the potential design choices that we could make. We identified eight design questions, with several choices to be made within them. Table 2 summarises these.

| **Questions** | **Choices** |
| --- | --- |
| - What list of news media domains to use? | - Own list, Alexa, Tranco, Cisco, Majestic |
| - How many news media domains to use? | - All domains, top 200, 100, 50, 20, 10 most visited |
| - What information to use within those domains? | - All URLs, only those identified as political |
| - What do we consider as being exposed to a URL? | - Visits equal or longer to 1 second, 30 seconds or 120 seconds |
| - What is the level of interest? | - Number of visits, number of minutes |
| - Should we use information from all devices? | - Mobile and PC, only PC, only mobile |
| - How many days of tracking should we use? | - 2, 5, 10, 15, 31 |
| - Should we use information from before or after the survey? | - Before, after, both |

**Table 2.** Design characteristics and choices for the concept “online news media exposure.”

Since it was unclear what design choices would be the ones yielding the most valid measures, we decided to apply our proposed approach to measure the validity of metered data measures (presented in section 6.1.1 of the paper). Hence, we created a variable for each potential combination, which resulted in 3,573 variables to measure the concept of online news media exposure.

**Analyses**

***Analysing convergent validity***

We first explored the convergent validity of the 3,573 variables computed. Convergent validity describes the fit between independent measures of the same underlying concept ([Prior, 2013](https://www.tandfonline.com/doi/full/10.1080/10584609.2013.819539)). Hence, if different variables were measuring the same underlying concept, they should highly correlate with each other. To explore whether this is the case or not, we computed one correlation for each potential pair of variables. Therefore, we obtained 6,349,266 unique Pearson’s correlation coefficients for each country.

***Analysing predictive validity***

Next, we explored the predictive validity of the different computed variables. Predictive validity refers to the degree to which a measurement instrument is related to a gold standard measurement ([Frey, 2008](https://methods.sagepub.com/reference/the-sage-encyclopedia-of-educational-research-measurement-and-evaluation/i16367.xml)). Measures closer to the theorised true relationship should be preferred. Often, when the true relationship is unknown, it is assumed that the higher the predictive power, the better. Although we do not make this assumption, any fluctuation in the predictive power of the variables would indicate differences in terms of predictive validity ([Prior, 2009](https://www.journals.uchicago.edu/doi/full/10.1017/S0022381609090781)). Specifically, we use political knowledge as gold measure, since it is the accepted practice in the media effects literature (see e.g., [Smith, Clifford and Jerit, 2020](https://journals.sagepub.com/doi/10.1177/1065912919882101); [Dilliplane, Goldman and Mutz, 2013](https://onlinelibrary.wiley.com/doi/full/10.1111/j.1540-5907.2012.00600.x); [Prior, 2009](https://www.journals.uchicago.edu/doi/full/10.1017/S0022381609090781)). To measure political knowledge, we use an additive political knowledge index that ranges from 0 to 4. Hence, for each of the 3,573 variables we ran an OLS regression model, with political knowledge as the dependant variable, the media exposure variable as the main independent variable, and some common control variables (age, gender, and educational level). Consequently, we obtained 3,573 partial regression coefficients for media exposure, in each country.

***Analysing the impact of each design choice on predictive validity***

Next, to understand the extent to which these choices affect the validity of measurements, drawing inspiration from the Survey Quality Predictor (SQP), we created a new dataset, in which the 3,573 variables were used as the observations, their associated partial regression coefficients as the dependant variables and the characteristics of the variables as the predictors. To predict the impact of each design choice, we used random forests of regression trees (R package randomForest) to extract the following information:

- the variable importance, measured as the percentage increase of Mean Squared Error (MSE) of the model if a specific variable had not been included in the trees used.

- the marginal effect of each design choices, understood as the adjusted predictions when holding all predictors constant.

**SOM 5 – Quantifying the prevalence and bias of tracking undercoverage**

This SOM discusses the approach followed to compute the prevalence of tracking undercoverage and simulate the potential bias it can introduce, focusing on the “average time spent on the Internet.”

**Analysing the prevalence of tracking undercoverage**

The prevalence of tracking undercoverage was estimated by combining survey questions and paradata Netquest collects from their metered panellists about the technology with which they were tracked, the type of device, the OS, whether it was a tablet or smartphone and, for plug-ins, the browser in which they were installed.

In terms of survey questions, we measured the number of devices used to access the Internet by asking the following: “During the last 15 days, from how many of these different types of devices have you accessed the Internet (including using apps such as Facebook, Twitter or YouTube)? Please, type the number of devices in the respective boxes.” The list of devices provided was designed to match the information available from the paradata. Specifically, we asked for: (1) Computer(s) with Windows OS; (2) Apple computer(s) (MAC); (3) Smartphone or tablet with Android OS; (4) Apple smartphone or tablet (iPhone or iPad); (5) Others.

Moreover, to assess the types of browsers used to access the Internet, we asked participants a maximum of three questions (depending on which devices were tracked, and the technology used according to the available paradata), as follows: “During the last 15 days, have you used any of the following web browsers to access the Internet through [a computer with Windows operating system/ an Apple computer (MAC)/ a smartphone or tablet with Android operating system]?” The list of browsers varied depending on the ones available in each OS.

Combining both sources of data, we created a variable indicating whether a participant had at least one device or browser not covered. (i.e., the number of devices and/or browsers tracked was lower than the self-reported one). Although we are mindful that self-reports might themselves be affected by measurement errors, we computed the proportion of individuals being undercovered, for each country in this way due to the lack of better information.

**Simulating the bias introduced by tracking undercoverage**

We used data from those participants being fully covered to run Monte Carlo simulations. Given that metered data was collected separately for computers and mobile devices, for fully covered participants it is possible to compute their estimates using all tracked targets ($F_{i}$), as well as only some of the tracked targets ($O_{i}$), simulating their estimates for specific undercoverage scenarios. Differences between the fully tracked behaviours and those affected by undercoverage ($Y_{i-}y_{i})$can be considered as tracking undercoverage bias. Hence, in our simulations we modified the targets used to compute the estimates and simulated the effect on those estimates for different tracking undercoverage scenarios. Specifically, to run the Monte Carlo simulations, we developed the following steps:

1. We identified the participants fully covered and tracked in both mobile and PC devices.
2. We defined seven tracking undercoverage scenarios to simulate varying the targets being omitted when computing the estimates (PCs or mobiles), and the prevalence in the sample. Specifically, we tested the effect of having 25%, 50% and 75 of participants with no PC or mobile device tracked. In addition, we conducted the simulation of the actual undercoverage scenario in our samples, together for PC and mobile.
3. For each of the coverage scenarios, we randomly created 1,000 allocation scenarios, in which all participants had the same chance to be undercovered. For instance, for the scenario with 25% of participants without any mobile device tracked, an individual would have .25 probability of having all the mobile devices untracked, and a .75 probability of being fully covered.
4. For each specific variable, in our case the “average time spent on the Internet”, 1,000 estimates were created for all seven tracking undercoverage scenarios. Participants selected as undercovered got part of their tracking data removed when computing the estimates of interest. As an example, if *Individual_i_* was tracked on a PC and a mobile device and *Individual_i_* was randomly selected to be non-tracked for all their mobile devices, all data from these devices would be considered as 0 when computing the estimates of interest. For the sake of simplicity, all complete losses of information were set to zero.
5. All estimates were computed using inverse probability weights created with the random forest relative frequency method (Buskrik and Kolenikov, 2015), to account for differences between the subsample of fully covered and the full sample of metered participants.
6. Finally, for each tracking undercoverage scenario, the average of the 1,000 simulations was considered as the average undercovered estimates. The difference between the average undercovered estimate and the fully covered estimate was considered as the average bias estimate.

As illustration, we focus on the “average time spent on the Internet”. First, we added the duration of the visits to all URLs and apps across all tracked devices and browsers to compute the total time spent on the Internet in a day. Then we computed the average time for the 15 days prior to the survey being answered.

**SOM 6 – Quantifying the prevalence of technology limitations**

Since we knew exactly what information could and could not be collected with each tracking technology (see SOM 1), as well as the specific technologies used to track each participant, we could compute the proportion of participants affected by the following technology limitations: 1) unobserved behaviours in incognito mode, 2) impossibility of tracking subdomain information, and 3) in-app information.

We computed the prevalence of all these limitations separately, for each country. We considered a participant to be affected by any of these limitations if at least one of their targets was tracked with a technology suffering from the limitation of interest.

**SOM 7 – Exploring whether participants tracked on iOS devices present different measurement properties**

We designed an approach to identify differential measurement properties for participants tracked on iOS devices. First, we asked participants the following: “Approximately, how much time do you spend on a typical day on the Internet (including using apps such as Facebook, Twitter or YouTube)? Please, type the number of hours and minutes in the respective boxes.”

We then combined this self-reported information with the observational data from the meter, to compute the absolute difference in minutes (Absolute difference: *|Self-reported time – Tracked time*|). Next, we ran a model exploring which variables were associated with this absolute difference. The main independent variable was “Tracked on iOS”, which indicates whether someone was tracked on an iOS or not (0 = No, 1 = Yes). The model also included other variables to control for potential confounders: general undercoverage (1= undercovered, 0 = fully covered); a self-reported measure of Internet use (continuous, minutes spent on the Internet on a typical day); the number of months that a participant had been part of the Netquest panels (continuous), as a proxy for panel loyalty; whether the person self-reported using mobile devices to access the Internet (0 = No, 1 = Yes); finally, we introduced age (continuous), gender (women= 0, male= 1) and whether a participant had completed higher education (0 = no, 1= yes). Although we expect both the survey and metered measures to be affected by errors, a significant effect of “Tracked on iOS” could indicate that participants tracked on an iOS present different measurement properties.

**SOM 8 – Quantifying the prevalence of participants with undercoverage-induced non-observations.**

We asked participants whether they had visited Twitter, Facebook, and the top 10 most visited news media domains in each country (according to Tranco: <https://tranco-list.eu/>) with non-tracked targets. Specifically, the questions asked: “During the last 15 days, have you used another device or browser apart from [INSERT DEVICE(S)] to visit the following web pages or apps.” For each individual, the devices inserted where those targets which we knew, thanks to the paradata, that participants were being tracked with. After that, the list of web pages / apps was presented, with a yes/no scale.

For each specific web pages and/or apps, participants were identified of having undercoverage-induced non-observations when they self-reported having visited those web pages and/or apps, but no behaviour had been tracked with the meters. Although we are mindful that the self-reports might also be affected by measurement errors, since we did not had access to other sources of information, after identifying those participants, we computed the proportion for each country.
